# Exploring the precision redox map during fasting-refeeding and satiation in *C. elegans*

**DOI:** 10.1007/s44154-023-00096-z

**Published:** 2023-06-12

**Authors:** Xinhua Qiao, Lu Kang, Chang Shi, Aojun Ye, Dongli Wu, Yuyunfei Huang, Minghao Deng, Jiarui Wang, Yuzheng Zhao, Chang Chen

**Affiliations:** 1 National Laboratory of Biomacromolecules, CAS Center for Excellence in Biomacromolecules, Institute of Biophysics, Chinese Academy of Sciences, Beijing, 100101 China; 2 School of Basic Medical Sciences of Southwest Medical University, Luzhou, 646000 China; 3 University of Chinese Academy of Sciences, Beijing, 100049 China; 4 School of Pharmacy, East China University of Science and Technology, Shanghai, 200237 China

**Keywords:** Redox, Fasting, Refeeding, Satiation, Hyperion, Grx1-roGFP2, *Caenorhabditis elegans*

## Abstract

Fasting is a popular dietary strategy because it grants numerous advantages, and redox regulation is one mechanism involved. However, the precise redox changes with respect to the redox species, organelles and tissues remain unclear, which hinders the understanding of the metabolic mechanism, and exploring the precision redox map under various dietary statuses is of great significance. Twelve redox-sensitive *C. elegans* strains stably expressing genetically encoded redox fluorescent probes (Hyperion sensing H_2_O_2_ and Grx1-roGFP2 sensing GSH/GSSG) in three organelles (cytoplasm, mitochondria and endoplasmic reticulum (ER)) were constructed in two tissues (body wall muscle and neurons) and were confirmed to respond to redox challenge. The H_2_O_2_ and GSSG/GSH redox changes in two tissues and three organelles were obtained by confocal microscopy during fasting, refeeding, and satiation. We found that under fasting condition, H_2_O_2_ decreased in most compartments, except for an increase in mitochondria, while GSSG/GSH increased in the cytoplasm of body muscle and the ER of neurons. After refeeding, the redox changes in H_2_O_2_ and GSSG/GSH caused by fasting were reversed in most organelles of the body wall muscle and neurons. In the satiated state, H_2_O_2_ increased markedly in the cytoplasm, mitochondria and ER of muscle and the ER of neurons, while GSSG/GSH exhibited no change in most organelles of the two tissues except for an increase in the ER of muscle. Our study systematically and precisely presents the redox characteristics under different dietary states in living animals and provides a basis for further investigating the redox mechanism in metabolism and optimizing dietary guidance.

**Supplementary Information:**

The online version contains supplementary material available at 10.1007/s44154-023-00096-z.

## Introduction

Cellular redox signaling is crucial for many aspects of physiological and pathological processes including different dietary strategies (Amigo and Kowaltowski 2014; Holmström and Finkel 2014). Fasting, refeeding and satiation are common states of life related to the diet, and metabolic pathways and redox reactions are inseparable in living organisms (Muri and Kopf 2021). Many studies have shown that dietary restriction (DR) could prolong the lifespan of rodents and nonhuman primates and improve health in humans (Green et al. 2022) and that redox regulation is one important mechanism. Clinical trials have also shown that fasting increases resistance to oxidative stress and reduces the incidence of aging-related diseases (Calabrese et al. 2010). The study of redox in different diets is important. However, the current research on redox and diet mostly focuses on total oxidant status and total antioxidant capacity but not on specific redox species or organelles. It is difficult for audience to understand the changes in redox status and metabolism.

The redox states after fasting, refeeding and satiation have been examined by researchers, although the studied states mostly pertained to the total redox or ex situ levels.

### The redox states after fasting

Many studies have reported that fasting can decrease hydrogen peroxide (H_2_O_2_) and reduced glutathione (GSH) levels. H_2_O_2_ produced by mitochondria isolated from the liver, skeletal muscle and brain in rats decreased after DR (Bevilacqua et al. 2004; Hagopian et al. 2005; Sanz et al. 2005; Caro et al. 2008). Electron transport chain complex I was inhibited and mitochondrial metabolism was suppressed during fasting, and the H_2_O_2_ release rate was lower than that of the control (Brown and Staples 2011). Compared with the random feeding group, the total H_2_O_2_ level and GSSG/GSH ratio of *C. elegans* in adults fed dilute bacteria decreased significantly (Back et al. 2012). Total GSH in the livers of mice was measured by commercially available kits and was decreased after fasting for 36 h (Abdelmegeed et al. 2009). The concentration of GSH decreased while GSSG increased in the livers of mice compared with the control after fasting (Balsam and Ingbar 1979; Grattagliano et al. 2003). The total GSH level in the lung was also reported to be decreased but no change was observed in mitochondria (Smith and Anderson 1992). However, research on the redox state after fasting in other species has different views. Research on *Chaos carolinensis* showed that basal respiration decreased progressively during starvation, and a spectrofluorometric assay based on the 2',7'-dichlorofluorescein diacetate dye indicated greater H_2_O_2_ and reactive oxygen species (ROS) generation in starved cells than in fed cells, and fasting increased oxidative stress (Deng et al. 2002). However, there was no difference between the intermittent fasting (IF) and control groups in isolated liver mitochondrial H_2_O_2_ production in mice (Carteri et al. 2021). Previous studies have reported inconsistent views on the redox state after fasting; in particular, redox changes at the organelle level are rarely reported.

### The redox states after refeeding

The studies showed that the redox changes brought about by fasting were reversed by refeeding. A study on mice showed that short-term refeeding after a 24-h starvation period restored H_2_O_2_ production back to normal levels in 3 h (Van den Branden et al. 1984). Another study reported the refeeding effects after fasting on the liver mitochondrial bioenergetics of snakes, and the results showed that the release of H_2_O_2_ increased in isolated mitochondria under basal respiration and that the oxidative phosphorylation capacity increased in the refeeding group compared to the fasting group (da Mota Araujo et al. 2021). Research on ducklings showed that fasting induced a decrease in the rates of oxidative phosphorylation, and maximal ROS release was completely reversed by 3 days of refeeding (Roussel et al. 2019). 2–3 days food-deprived rats had a significantly higher liver GSSG/GSH ratio, and refed rats had significantly greater liver GSH levels than unfed rats (Leeuwenburgh and Ji 1996, Jonas et al. 1999). Some limitations are that these studies were not performed in situ, and we are not clear about the actual situation in living organisms.

### The redox states after satiation

There are few direct measurements of redox states and descriptions of satiation, and we refer to some relevant studies. A study showed that ROS are the molecular actors involved in brain nutrient sensing. Inhibition of the production of ROS stimulated by hypertriglyceridemia in rats completely eliminated symptom-related satiety (Benani et al. 2007). Proopiomelanocortin (POMC) neurons were activated to enhance satiety and significantly reduce food intake after intraperitoneal injection of H_2_O_2_. It is meaningful to study the redox state under dietary satiety which is related to metabolism.

Experts provide guidelines for measuring ROS and oxidative damage in cells and in vivo because the application and interpretation of redox measurements are fraught with challenges and limitations (Murphy et al. 2022), and accurately evaluating the redox state under different situations is the premise for comprehending its specific role. We emphasize that the description of redox status must be considered in the context of species, time, place, level, and target (Meng et al. 2021). Research on the precise regulation of metabolism and redox balance is urgently needed to provide clarity and breakthroughs. Metabolic pathways and redox reactions are the core of cell function. Cytoplasm, mitochondria, and ER are the main cell organelles involved in glucose and fat metabolism, and the exchanges of metabolites and redox small molecules frequently occur between them. Redox and metabolism in the muscle and nervous system are associated with various neurodegenerative diseases, such as PD and ALS. Therefore, we have preferentially selected these organelles and tissues for study. In this study, we provide precise redox descriptions during fasting, refeeding and satiation. We utilized the ratiometric redox probe Hyperion sensing H_2_O_2_ and Grx1-roGFP2 sensing GSH/GSSG located in the cytoplasm, mitochondria and ER of the body wall muscle and neurons to detect the redox status of *C. elegans* during fasting, refeeding and satiation and provide a precision redox map of *C. elegans* in vivo under different dietary conditions, as shown by genetically encoded redox probes.

## Results

### Validation of the localization and redox response of the cyto-Hyperion, mito-Hyperion and ER-Hyperion in body muscle and neurons in *C. elegans*

The main sources of ROS in the cytoplasm include NADH oxidase (Nox) 1/2/4 on the cell membrane (Lambeth et al. 2007) and a large number of xanthine oxidases catalyzing reactions in the cytoplasm (Cantu-Medellin and Kelley 2013). Energy metabolism in the mitochondria is associated with the production of ROS. Electron transport through the mitochondrial respiratory chain is efficient, while 1–2% of electrons are leaked to generate O_2_^−^ in reactions mediated by coenzyme Q and its complexes (Kamata and Hirata 1999). In the ER, H_2_O_2_ is produced through oxidative folding. NADPH oxidase 4 produces H_2_O_2_ over superoxide on the ER membrane. ER oxidoreductin 1 (ERO1) is estimated to consume 25% of the oxygen (O_2_) available in the cell, and ERO1 reoxidizes PDI and reduces O_2_ to H_2_O_2_ (Konno et al. 2021).

**Fig. 1:**
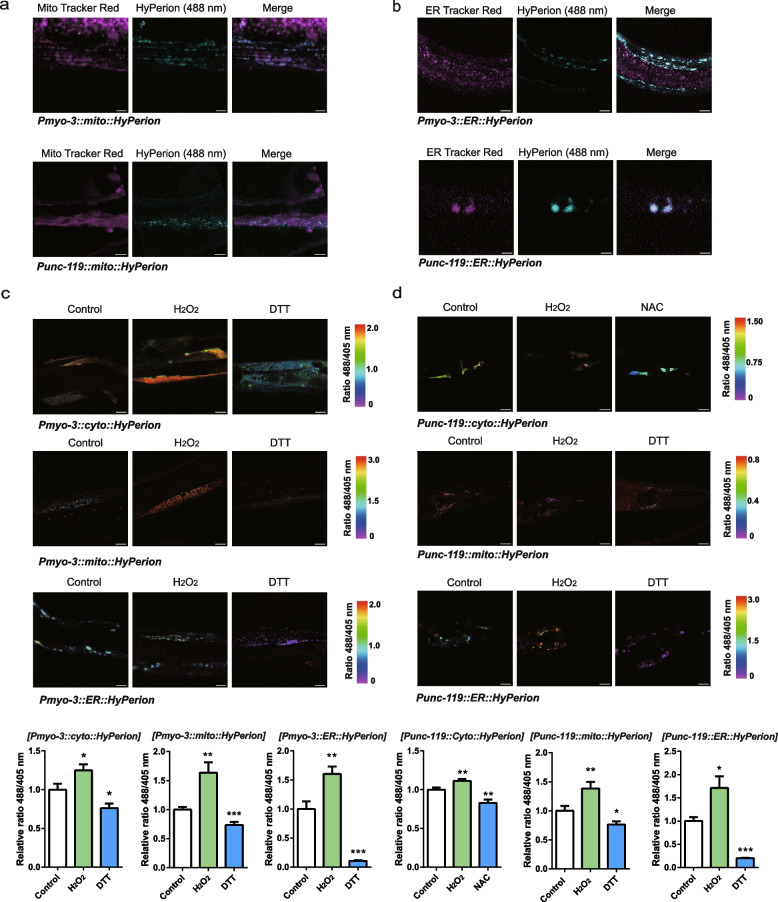
Localization and redox response of the cyto-Hyperion, mito-Hyperion and ER-Hyperion in body muscle and neurons in *C. elegans.***a**, **b** Location of mito-Hyperion and ER-Hyperion. Confocal imaging of mito-Hyperion (488 nm) with MitoTracker Red (594 nm) and ER-Hyperion (488 nm) with ER tracker Red (594 nm) in the body muscle and neurons of *C. elegans*. *myo-3p::mito::Hyperion* (bar = 5 μm), *unc-119p::mito::Hyperion* (bar = 4 μm), *myo-3p::ER::Hyperion* (bar = 15 μm), and *unc-119p::ER::Hyperion* (bar = 3 μm). **c**, **d** The relative 488/405 nm ratio of cyto-Hyperion, mito-Hyperion and ER-Hyperion in body muscle and neurons treated with DTT (or NAC) and H_2_O_2_ at 500 ~ 550 nm was measured with confocal microscopy. *myo-3p::cyto::Hyperion* (*n* = 12) (1 mM H_2_O_2_, 5 mM DTT, bar = 15 μm), *myo-3p::mito::Hyperion* (*n* = 15) (20 μm H_2_O_2_, 1 mM DTT, bar = 15 μm), *myo-3p::ER::Hyperion* (Control, *n* = 21; H_2_O_2_, *n* = 26, DTT, *n* = 13) (1 mM H_2_O_2_, 1 mM DTT, left panel bar = 30 μm, right panel bar = 15 μm), *unc-119p::cyto::Hyperion* (*n* = 20) (20 μm H_2_O_2_, 1 mM NAC, bar = 15 μm), *unc-119p::mito:: Hyperion* (Control, *n* = 20; H_2_O_2_, *n* = 15, DTT, *n* = 15) (1 mM H_2_O_2_, 1 mM DTT, bar = 15 μm), and *unc-119p::ER:: Hyperion* (*n* = 10) 1 mM H_2_O_2_, 1 mM DTT, bar = 15 μm). Data are shown as the mean ± SEM. *p < 0.05, **p < 0.01, ***p < 0.001 by unpaired t test

The Hyperion probe responded well to H_2_O_2_, which was measured at 500 ~ 550 nm after excitation at 405 and 488 nm by confocal microscopy, the ratio of 488/405 nm indicated the relative level of H_2_O_2_. We obtained the *C. elegans* strains incorporating Hyperion by microinjection. *mito-Hyperion* and *ER-Hyperion* were generated using *Hyperion* cDNA as a template by adding mitochondrial or ER retention sequences. Then, we constructed plasmid cyto-Hyperion, mito-Hyperion and ER-Hyperion vectors in L2534 (located in the body wall muscles) and pPD49.26 (located in the neurons) and obtained six stable *C. elegans* strains *Pmyo-3::cyto-Hyperion*, *Pmyo-3::*mito-*Hyperion*, *Pmyo-3::ER-Hyperion, unc119:: cyto-Hyperion*, *unc119::*mito-*Hyperion* and *unc119::ER-Hyperion* by microinjection. Mito-Hyperion and ER-Hyperion in body muscle and neurons colocalized with the mitochondrial tracker and ER tracker (Fig. 1a and b), indicating their localization in mitochondria and the ER, respectively. Cyto-Hyperion, mito-Hyperion and ER-Hyperion in body muscle and neurons responded well to reductive challenge with dithiothreitol (DTT) or NAC and oxidative challenge with H_2_O_2_ (Fig. 1c and d).

### Relative H_2_O_2_ levels during fasting, refeeding and satiation in three organelles within two tissues in *C. elegans*

*C. elegans* is an ideal model for studying feeding behavior due to its small and delicate nervous system and anatomical features. *C. elegans* and mammals share common mechanisms of signal transduction, metabolic pathways and even information processing in energy homeostasis and fat metabolism (Davis et al. 2017). We then imaged the cyto-Hyperion, mito-Hyperion and ER-Hyperion in body muscle and neurons using confocal microscopy to test the redox state under fasting, refeeding and satiation. Fasting of *C. elegans* was verified using Oil Red O staining under a microscope after 12 h of fasting. Except for the eggs, the fat in other parts, such as the pharynx and body wall muscles, was consumed (Fig. S1a). After 12 h of dietary restriction, the lifespan of *C. elegans* was significantly increased compared with that of the wild type (Fig. S1b), which indicated that the fasting model worked well. There are fixed behaviors in mammals that characterize satiety: they stop eating, tidy up to explore for a short period of time, and then rest or sleep (Antin et al. 1975). Similar behaviors were found in *C. elegans,* which exhibited temporary cessation of movement and food intake after satiation (You et al. 2008). We picked worms that remained stationary for 10 s and whose pharynx had stopped pumping for redox assays (Fig. S1c).

**Fig. 2:**
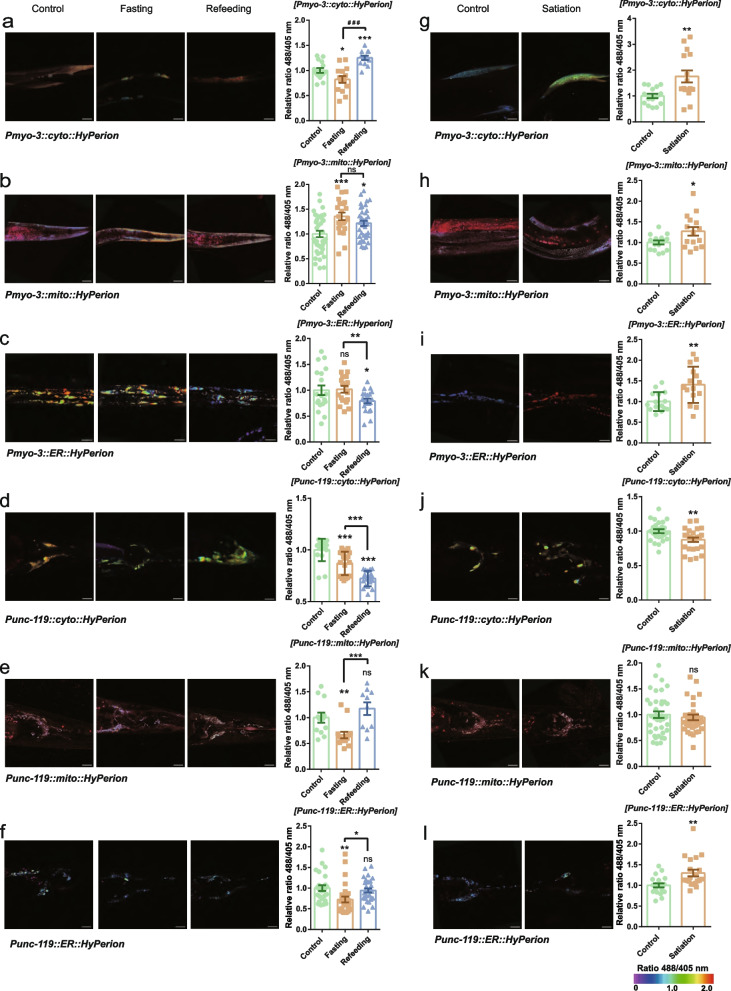
Relative H_2_O_2_ levels during fasting, refeeding and satiation in three organelles within two tissues in *C. elegans*. **a**, **b**, **c**, **d**, **e**, **f** The relative 488/405 nm ratio of cyto-Hyperion, mito-Hyperion and ER-Hyperion in body muscle and neurons of *C. elegans* during fasting and refeeding*. myo-3p::cyto::Hyperion* (Control, *n* = 15; Fasting, *n* = 14; Refeeding, *n* = 12) (bar = 24 μm), *myo-3p::mito::Hyperion* (Control, *n* = 39; Fasting, *n* = 22; Refeeding, *n* = 37) (bar = 30 μm), *myo-3p::ER::Hyperion* (Control, *n* = 19; Fasting, *n* = 18; Refeeding, *n* = 21) (bar = 25 μm), *unc-119p::cyto::Hyperion* (Control, *n* = 17; Fasting, *n* = 22; Refeeding, *n* = 19) (bar = 15 μm), *unc-119p:::mito::Hyperion* (Control, *n* = 12; Fasting, *n* = 14; Refeeding, *n* = 10) (bar = 15 μm), and *unc-119p:::ER::Hyperion* (Control, *n* = 25; Fasting, *n* = 27; Refeeding, *n* = 29) (bar = 15 μm). **g**, **h**, **i**, **j**, **k**, **l** The relative 488/405 nm ratio of cyto-Hyperion, mito-Hyperion and ER-Hyperion in body muscle and neurons of *C. elegans* during satiation of *C. elegans. myo-3p::cyto::Hyperion* (*n* = 15) (bar = 15 μm), *myo-3p::mito::Hyperion* (*n* = 15) (bar = 25 μm), *myo-3p::ER: Hyperion* (*n* = 15) (left: 25 μm, right: 50 μm), *unc-119p::cyto::Hyperion* (*n* = 25) (bar = 15 μm), *unc-119p:::mito::Hyperion* (*n* = 38) (bar = 50 μm), and *unc-119p::::ER::Hyperion* (*n* = 20) (bar = 15 μm). Data are shown as the mean ± SEM. *p < 0.05, **p < 0.01, ***p < 0.001 by unpaired t test

In the cytoplasm of body muscle, the relative level of H_2_O_2_ was markedly decreased after fasting compared with the control but markedly increased after refeeding compared with the control or fasting group (Fig. 2a). The H_2_O_2_ level increased markedly after fasting and refeeding compared with the control in the mitochondria of body muscle (Fig. 2b). The H_2_O_2_ level showed no change in the ER after fasting but decreased markedly after refeeding compared with the control or fasting group (Fig. 2c). Neurons within the central nervous system are extremely sensitive to changes in the pressure of oxygen and energy due to their high energetic demand (Peña and Ramirez 2005). In the cytoplasm of neurons, the relative level of H_2_O_2_ was markedly decreased after fasting and refeeding compared with the control, and it was lower in the refeeding group than in the fasting group (Fig. 2d). The H_2_O_2_ level decreased markedly after fasting but showed no change after refeeding compared with the control in the mitochondria of neurons (Fig. 2e). It is noteworthy that the H_2_O_2_ level increased markedly in the refeeding group compared with the control group, indicating that refeeding after starvation could cause a large explosion of ROS generation, which may be the reason why antioxidant enzymes were significantly highly expressed after refeeding compared with expression in the control (Honma et al. 2020). In the ER of neurons, the relative level of H_2_O_2_ was markedly decreased after fasting and showed no change after refeeding compared with the control. The H_2_O_2_ level increased markedly in the refeeding group compared with the fasting group, showing that food supplementation increased the H_2_O_2_ level and returned it to the control level in the ER (Fig. 2f). Under the satiation state, the relative level of H_2_O_2_ was markedly increased in the cytoplasm, mitochondria and ER of body muscle compared with levels in the control (Fig. 2g, h and i), which may be closely related to the metabolic level. Glucagon-like peptide-1 (GLP-1) is a potent satiating hormone in response to eating, and dietary fat, in particular monounsaturated fatty acids, potently stimulates GLP-1 secretion from L-cells and increases cellular respiration (Clara et al. 2016). In neurons, the relative level of H_2_O_2_ was markedly decreased in the cytoplasm, while there was no change in mitochondria in the satiation state, but the level was increased in the ER compared with the control (Fig. 2j, k and l).

### Validation of the localization and redox response of cyto-Grx1-roGFP2, mito-Grx1-roGFP2 and ER-sf-roGFP in body muscle and neurons in *C. elegans*

Glutathione (L-γ-glutamyl-cysteinyl-glycine) is a tripeptide synthesized through consecutive enzymatic reactions, and the ratio of reduced glutathione (GSH) to oxidized glutathione (GSSG), which characterizes the cellular redox status, is approximately 100/1 in the cytoplasm, 10/1 in mitochondria, and 3/1 to 1 in the ER (Kalinina and Novichkova 2021). The glutathione system mainly includes glutathione, glutathione peroxidases (GPxs), glutathione reductase (GR), glutathione S-transferases (GSTs) and glutaredoxins (Grxs) (Go and Jones 2010). The system uses GSH and NADPH as reducing equivalents, removes ROS and completes the recycling of GSH through GPxs and GR while reducing protein sulfhydryl groups. The ratio of GSH/GSSG also affects the level of NADH/NAD^+^ in the mitochondria (Cortés-Rojo et al. 2020).

**Fig. 3:**
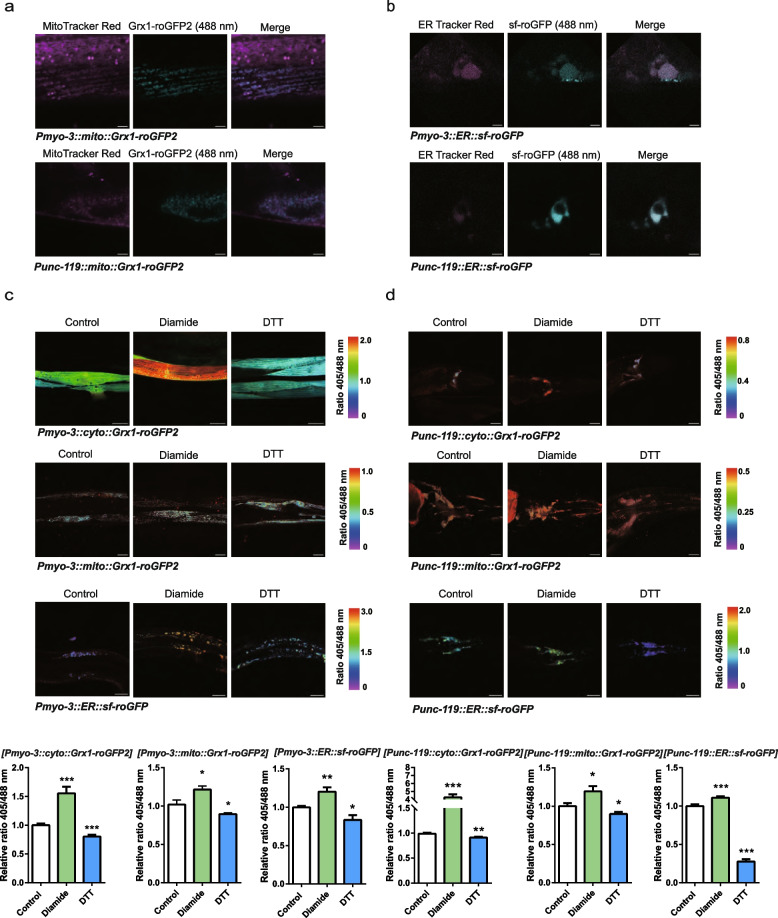
Localization and redox response of cyto-Grx1-roGFP2, mito-Grx1-roGFP2 and ER-sf-roGFP in the body muscle and neurons in *C. elegans*. **a**, **b** Location of mito-Grx1-roGFP2 and ER-sf-roGFP. Confocal imaging of mito-Hyperion (488 nm) with MitoTracker Red (594 nm) and ER-Hyperion (488 nm) with ER tracker Red (594 nm) in body muscle and neurons of *C. elegans*. *myo-3p::mito::Grx1-roGFP2* (bar = 5 μm), *unc-119p::mito::Grx1-roGFP2* (bar = 5 μm), *myo-3p::ER::sf-roGFP2* (bar = 5 μm), and *unc-119p::ER::sf-roGFP2* (bar = 3 μm). **c**, **d** The relative 405/488 nm ratio of cyto-Grx1-roGFP2, mito-Grx1-roGFP2 and ER-sf-roGFP in body muscle and neurons treated with 1 mM diamide and 1 mM DTT at 500 ~ 550 nm was measured with confocal microscopy. *myo-3p::cyto::Grx1-roGFP2* (*n* = 13) (bar = 15 μm, 5 mM DTT), *myo-3p::mito::Grx1-roGFP2* (*n* = 17) (bar = 15 μm, 5 mM DTT)), *myo-3p::ER::sf-roGFP* (*n* = 10) (bar = 30 μm), *unc-119p::cyto::Hyperion* (*n* = 15) (bar = 24 μm), *unc-119p::mito::Grx1-roGFP2* (*n* = 10) (bar = 15 μm), and *unc-119p::ER::sf-roGFP* (Control, *n* = 25; Diamide, *n* = 25; DTT, *n* = 13) (bar = 30 μm). Data are shown as the mean ± SEM. *p < 0.05, **p < 0.01, ***p < 0.001 by unpaired t test

The Grx1-roGFP2 and sf-roGFP probes responded well to GSSG/GSH, which were measured at 500 ~ 550 nm after excitation at 405 and 488 nm by confocal microscopy, and the ratio of incorporated 405/488 nm indicated the relative level of GSSG/[GSH]^2^. We obtained *C. elegans* strains incorporating Grx1-roGFP2 and sf-roGFP by microinjection. *mito-Grx1-roGFP2* and *ER-sf-roGFP* were generated using *Grx1-roGFP2* and *sf-roGFP* cDNA as templates by adding mitochondrial or ER retention sequences. Then, we constructed the plasmids *Grx1-roGFP2*, mito-*Grx1-roGFP2* and *sf-roGFP* in the L2534 and pPD49.26 vectors, respectively, and obtained six stable *C. elegans* strains, namely, *Pmyo-3::cyto-Grx1-roGFP2*, *Pmyo-3::*mito-*Grx1-roGFP2*, *Pmyo-3::ER-sf-roGFP, unc119::cyto-Grx1-roGFP2*, *unc119::*mito-*Grx1-roGFP2* and *unc119::ER-sf-roGFP* by microinjection. mito-Grx1-roGFP2 and ER-*sf-roGFP* in body muscle and neurons colocalized with the mitochondrial tracker and ER tracker (Fig. 3a and b), indicating their localization in mitochondria and the ER, respectively. Grx1-roGFP2, mito-Grx1-roGFP2 and ER-sf-roGFP in body muscle and neurons responded well to reductive challenge with DTT and oxidative challenge with diamide (Fig. 3c and d).

### Relative GSSG/GSH levels during fasting, refeeding and satiation in three organelles within two tissues in *C. elegans*

**Fig. 4:**
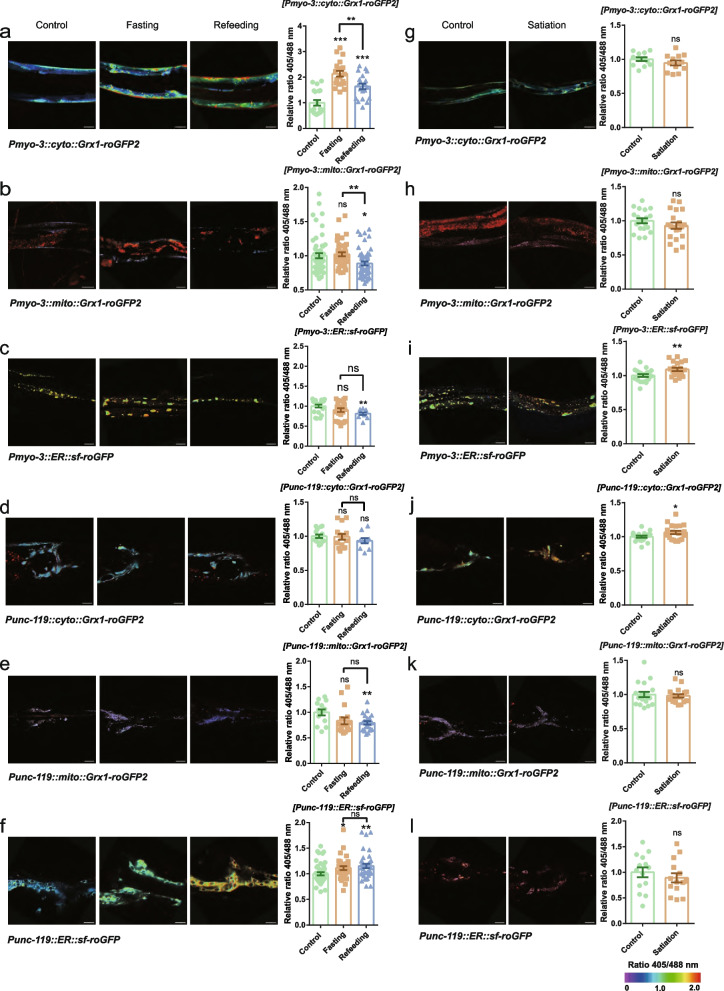
Relative GSSG/GSH levels during fasting, refeeding and satiation in three organelles within two tissues in *C. elegans*. **a**, **b**, **c**, **d**, **e**, **f** The relative 405/488 nm ratio of cyto-Grx1-roGFP2, mito-Grx1-roGFP2 and ER-sf-roGFP in body muscle and neurons of *C. elegans* during fasting and refeeding*. myo-3p::cyto::Grx1-roGFP2* (*n* = 20) (bar = 50 μm), *myo-3p::mito::Grx1-roGFP2* (Control, *n* = 54; Fasting, *n* = 44; Refeeding, *n* = 51) (bar = 30 μm), *myo-3p::ER::sf-roGFP* (Control, *n* = 21; Fasting, *n* = 25; Refeeding, *n* = 10) (bar = 15 μm), *unc-119p::cyto::Grx1-roGFP2* (Control, *n* = 15; Fasting, *n* = 15; Refeeding, *n* = 10) (bar = 15 μm), *unc-119p:::mito::Grx1-roGFP2* (Control, *n* = 14; Fasting, *n* = 16; Refeeding, *n* = 23) (bar = 15 μm), and *unc-119p::::ER::sf-roGFP* (Control, *n* = 43; Fasting, *n* = 33; Refeeding, *n* = 35) (bar = 8 μm). **g**, **h**, **i**, **j**, **k**, **l** The relative 405/488 nm ratio of cyto-Grx1-roGFP2, mito-Grx1-roGFP2 and ER-sf-roGFP in body muscle and neurons of *C. elegans* during satiation*. myo-3p::cyto::Grx1-roGFP2* (*n* = 12) (bar = 50 μm), *myo-3p::mito::Grx1-roGFP2* (*n* = 20) (bar = 25 μm), *myo-3p::ER::sf-roGFP* (*n* = 20) (bar = 15 μm), *unc-119p::cyto::Grx1-roGFP2* (*n* = 19) (bar = 15 μm), *unc-119p:::mito::Grx1-roGFP2* (*n* = 19) (bar = 15 μm), and *unc-119p::ER::sf-roGFP* (*n* = 14) (bar = 15 μm). Data are shown as the mean ± SEM. *p < 0.05, **p < 0.01, ***p < 0.001 by unpaired t test

In the body muscle, the relative level of GSSG/GSH was increased markedly after fasting and refeeding compared with the control in the cytoplasm, and it was lower in the refeeding group than in the fasting group (Fig. 4a). The GSSG/GSH level showed no change after fasting but decreased markedly after refeeding compared with the control or fasting group in mitochondria (Fig. 4b). The GSSG/GSH level showed no change after fasting in the ER (Fig. 4c). In the cytoplasm of neurons, the GSSG/GSH level showed no change among the control, fasting and refeeding groups (Fig. 4d). The GSSG/GSH level had a downward trend but was not significantly different in the mitochondria of neurons after fasting but decreased markedly after refeeding compared with the control, and there was no change between the fasting and refeeding groups (Fig. 4e). In the ER of neurons, the relative level of GSSG/GSH increased markedly after fasting and refeeding compared with the control, and there was no change between the fasting and refeeding groups (Fig. 4f). Under the satiation state, the relative level of GSSG/GSH increased markedly in the ER of body muscle but exhibited no change in the other organelles of body muscle and neurons compared with the control (Fig. 4g, h, i, j, k and l).

### Precision redox map of *C. elegans* during refeeding and satiation

**Fig. 5:**
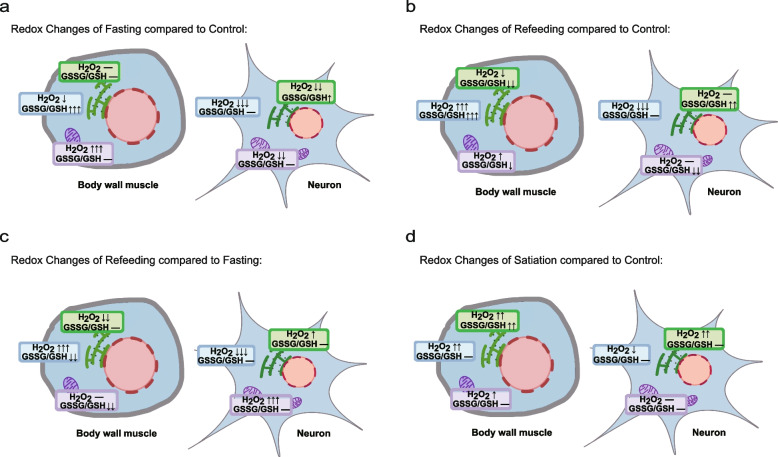
Summary of the redox map of *C. elegans* during fasting, refeeding and satiation. **a** The redox map of *C. elegans* during fasting. **b** The redox map of *C. elegans* during refeeding compared to the control. **c** The redox map of *C. elegans* during refeeding compared to fasting. **d** The redox map of *C. elegans* during satiation

In summary, under fasting conditions, the relative level of H_2_O_2_ decreased significantly in most parts, except that H_2_O_2_ increased markedly in mitochondria and did not change in the ER of body muscle. The relative level of H_2_O_2_ decreased in the cytoplasm but increased in the mitochondria of the body muscle. The relative level of H_2_O_2_ in the cytoplasm, mitochondria and ER of neurons decreased markedly, implying that these cells may be in a state of reductive stress. GSSG/GSH showed no significant changes in most parts but increased significantly in the cytoplasm of body muscle and the ER of neurons. The relative level of GSSG/GSH in the cytoplasm of body muscle was increased markedly but showed no change in the mitochondria and ER. While the relative level of GSSG/GSH in the ER of neurons was markedly increased, the cytoplasm and mitochondria of neurons showed no changes (Fig.5a). The maintenance of the high oxidative power of the ER is important for its function. Higher levels of GSSG/GSH in the ER after fasting may contribute to the functional improvement of the ER.

After refeeding, most redox species levels of H_2_O_2_ and GSSG/GSH in different tissues and organs returned to the control state. Some of them had reached normal levels, which means that they were not different from the control, such as the relative level of H_2_O_2_ in the mitochondria and ER of neurons. Some of them revealed that they had undergone a significant reverse change compared with fasting but were still significantly different from the control, such as the relative level of H_2_O_2_ in the mitochondria of body wall muscle. The GSSG/GSH ratio in mitochondria of body wall muscle and neurons did not change after fasting, but the ratio in both tissues decreased after refeeding (Fig. 5b). We also systematically compared fasting and refeeding, as representative models of consuming food after starvation in daily life. In the body muscle, the relative level of H_2_O_2_ showed no change in mitochondria but decreased markedly in the ER in the refeeding group compared with the fasting group. The relative GSSG/GSH level decreased markedly in the cytoplasm and mitochondria in the refeeding group compared with the fasting group. The cells were in a state of reductive stress in the refeeding group compared with the fasting group. In the neurons, the relative level of H_2_O_2_ in the mitochondria and ER increased markedly in the refeeding group compared with the fasting group, indicating that cells were in a more oxidative state after refeeding (Fig. 5c).

In the satiated state, the relative level of H_2_O_2_ in the cytoplasm, mitochondria and ER of the body muscle was markedly increased. In neurons, the relative level of H_2_O_2_ was decreased in the cytoplasm and increased in the ER, while there was no change in mitochondria compared with the control. In body muscle, the relative levels of GSSG/GSH were not changed in the cytoplasm and mitochondria but increased in the ER compared with the control. In neurons, the GSSG/GSH ratio was not changed in the cytoplasm, mitochondria or ER (Fig. 5d). In summary, mitochondria and ER in the body wall muscle and ER in neurons of *C. elegans* became more oxidized, but there was no change in the cytoplasm of the body wall muscle and mitochondria in neurons.

## Discussion

In this study, we first measured an in situ real-time and dynamic precision redox map under three dietary conditions (fasting, refeeding and satiation) at the organelle level (cytoplasm, mitochondria and ER) based on two redox species (H_2_O_2_ and GSH) in two tissues (body wall muscle and neurons) using *C. elegans* as a model. We have achieved a precise description of the redox state with respect to a specific “species”, “place” (organelle and tissue), “time” (fasting, refeeding and satiation) and “level” for the first time. In particular, the redox state of the ER under different dietary conditions has not been reported. Different dietary states affect redox homeostasis in nematodes in tissue-specific and organelle-specific ways. These findings suggest that precision redox characteristics should be considered in future studies of diet and metabolism. The diverse changes in redox in various organelles and tissues may provide a new perspective for future precision redox medicine.

Metabolic pathways and redox reactions are the core of cell function (Muri and Kopf 2021). In view of the critical role of the redox system in metabolic pathways, it is important to precisely describe the redox species generated in cellular metabolism, which may be essential for different cellular signaling or physiological functions, and this in situ and precision redox map of *C. elegans* during fasting proved this point. Our previous studies showed that the ER undergoes reductive stress during senescence and that ER reductive stress accelerates senescence in human cells and *C. elegans* (Qiao et al. 2022), and exercise alleviates age-related ER reductive stress and delays aging (Meng et al. 2021). We described the redox changes in the ER after fasting for the first time. We found that GSSG/GSH increased in the ER of neurons after fasting, indicating that the ER was in a more oxidative state. This result suggested that fasting was one way to maintain the oxidation power of the ER, which may contribute to its effect of delaying aging. Our results showed that H_2_O_2_ increased significantly in the mitochondria of body muscle. ROS could promote mammalian transcription factor ATF5 trafficking (its homologous gene is ATFS-1 in *C. elegans*) to the nucleus, where it activates the mitochondrial UPR (UPR^mt^) (Fiorese et al. 2016). UPR^mt^ activation promotes lifespan extension (Shpilka and Haynes 2018). A study also showed that DR promotes longevity in *C. elegans* by maintaining mitochondrial network homeostasis (Weir et al. 2017). We can reasonably infer that mitochondrial H_2_O_2_ produced by fasting plays an important role in promoting longevity. Our results also showed that H_2_O_2_ decreased significantly in most parts of the body muscle and neurons after fasting, which was consistent with a previous report on total redox or isolated mitochondria levels (Wilhelmi de Toledo et al. 2020; Honjoh et al. 2009). H_2_O_2_ in the mitochondria of neurons was decreased after fasting, which is worth considering. It has been reported that intermittent fasting results in tissue-specific changes in the redox state of rats and that intermittent fasting leads to the redox imbalance of the liver and brain but protects the heart from oxidative damage (Chausse et al. 2015). The changes in different redox species in the same organelle under the same conditions were inconsistent or even opposite, emphasizing the importance of accurate redox descriptions.

The overall redox changes brought by refeeding have been studied, while our study was still at the forefront of the research at the organelle level. Previous studies showed that the redox changes caused by fasting were reversed by refeeding, consistent with our study. A study of short-term starvation and refeeding on glutathione in skeletal muscles showed that glutamate and glutamine decreased after starvation, while these changes returned to normal levels during the refeeding period (Hammarqvist et al. 2005). GSSG/GSH in the mitochondria of body wall muscle and neurons did not change after fasting, but both decreased after refeeding, and a study on the redox state in refed rats supported our conclusions (Leeuwenburgh and Ji 1996).

There are few reports about redox in the satiety state. We first systematically provide an in situ redox atlas during satiation concerning two redox species in three organelles of two tissues, and this study fills the gap in this field. Our results showed that the mitochondria and ER in the body wall muscle and the ER in neurons of *C. elegans* became more oxidized, which was supported by a previous report (Benani et al. 2007). The increase in ROS levels in the brains of mice activated preopiomelanocortin neurons, increased the sense of satiety and reduced food intake (Diano et al. 2011). It is now understood that ROS play an important role in the control of food intake, in which modifications in mitochondrial dynamics in either agouti-related peptide (AgRP) or proopiomelanocortin (POMC) circuits that regulate feeding behavior are related to energy balance (Lahera et al. 2017), and energy metabolism is associated with the production of reactive oxygen species (ROS) that are in turn connected with the feeding/fasting cycle (Mezhnina et al. 2022). An important source of H_2_O_2_ is the ER protein folding process (Rashdan and Pattillo 2020), and our study showed that the H_2_O_2_ level in the ER of both tissues increases significantly during satiety, which may be due to the increased protein synthesis activity at this stage.

We presented the redox characteristics under different dietary states in living animals. It is currently not completely clear how redox mediates these processes. The upstream signals that regulate redox changes under different dietary states also remain to be determined, as does whether this regulation is conserved in mammals, and it is worth validating the current findings in mammals, and the precision redox map under different dietary states needs to be studied in primates. Moreover, the precise mechanism and physiological relevance of redox regulation during fasting, refeeding and satiation need to be explored. Sies et al*.* suggested that the horizon for redox precision medicine is opening (Sies and Jones 2020). If these questions can be answered, the diverse changes in redox in various organelles and tissues may provide a new perspective for redox mechanism in metabolism and optimizing dietary guidance.

## Materials and methods

### *C. elegans* strains and culture

The *C. elegans* strains used in this study were Bristol N2 (obtained from the *Caenorhabditis elegans* Genetics Center). Extrachromosomal transgenic strains were obtained by microinjection. A total of 100 ng/μL transgene plasmid was injected into the gonads of *C. elegans.* The extrachromosomal arrays were integrated by exposing the animals to γ-irradiation that were subsequently backcrossed three times. The strains we obtained were as follows: *Is[myo-3p::cyto::Hyperion]*, *Is[myo-3p::mito::Hyperion]*, *Is[myo-3p::ER::Hyperion]*, *Is[myo-3p::cyto::Grx1-roGFP2], Is[myo-3p::mito::Grx1-roGFP2], Is[myo-3p::ER:sf-roGFP], Is[unc-119p::cyto::Hyperion]*, *Is[unc-119p::mito::Hyperion]*, *Is[unc-119p::ER::Hyperion]*, *Is[unc-119p::cyto::Grx1-roGFP2], Is[unc-119p::mito::Grx1-roGFP2],* and *Is[unc-119p::ER::sf-roGFP].*

The *C. elegans* strains used in this study were maintained at 20 °C on standard nematode growth media seeded with the OP50 or HB101 strain of *Escherichia coli* as their food source.

### Fasting, refeeding and satiation model

Fasting model: *C. elegans* adults on Day 1 were starved for 12 h. Refeeding model: starved *C*. *elegans* were refed with *E. coli* OP50 for 1 h, and *C. elegans* fed with *E. coli* OP50 was used as the control. Satiation model: *C. elegans* fed with *E. coli* HB101 were starved for 12 h, and the starved *C*. *elegans* were refed with *E. coli* HB101 until the *C*. *elegans* remained stationary for 10 s and their pharynx stopped pumping. *C. elegans* fed *E. coli* HB101 was used as the control.

### Determination of the *C. elegans* redox state with confocal microscopy

Redox states were detected using confocal microscopy as described previously (Wang et al. 2022), and individual cells of 20 animals were analyzed for each condition. In brief, images were taken on a Zeiss LSM710 confocal microscope using a 63 × objective. Live nematodes were excited with 405 and 488 nm lasers, and the emission was detected from 500 to 530 nm. Images were analyzed using Zen (Zeiss) and ImageJ (National Institutes of Health) software.

Nematodes with the redox reporter (Hyperion and Grx1-roGFP2) were treated with 10 mM DTT or 1 mM H_2_O_2_ or diamide as a positive control of the probe response to redox.

### Confocal microscopy confirmation of subcellular localization

Worms (*Is[myo-3p::mito::Hyperion]*, *Is[myo-3p::ER::Hyperion]*, *Is[myo-3p::mito::Grx1-roGFP2], Is[myo-3p::ER:sf-roGFP], Is[unc-119p::mito::Hyperion]*, *Is[unc-119p::ER::Hyperion], Is[unc-119p::mito::Grx1-roGFP2]*, and *Is[unc-119p::ER::sf-roGFP])* were exposed for 24 h to 10 μM ER-Tracker Red or mito-Tracker Red at 20 ℃. Following 10 min of intestinal clearance of fluorescent dyes on NGM agar plates, living nematodes were reversibly paralyzed on glass slides with levamisole, and confocal microscopy was used to confirm subcellular fluorescence localization.

### Research schematic of the precision redox map of *C. elegans* during fasting, refeeding and satiation

To study the precision redox map of *C. elegans* during fasting, refeeding and satiation, we utilized the ratiometric redox probe Hyperion sensing H_2_O_2_ (Meng et al. 2021) and the Grx1-roGFP2 sensing GSH/GSSG couple (Qiao et al. 2022) to detect the redox status of *C. elegans*. We stably overexpressed these two redox probes in the cytoplasm, mitochondria and ER of the body wall muscle and neurons in *C. elegans* and obtained 12 transgenic *C. elegans* with redox fluorescent probes. We confirmed the localization of Hyperion and Grx1-roGFP2 in different tissues and organelles and whether they would correctly respond to redox stimuli. Then, a precision redox map of *C. elegans* during fasting, refeeding and satiation was generated by confocal microscopy (Figs. S2a and 2b). Fasting is considered to be a way to improve health. C*. elegans* were starved for 12 h from Day 1, and then the starved *C*. *elegans* were refed with *E. coli* OP50 for 1 h, which was used as the refeeding model (Fig. S2c). The redox map in satiety has never been reported. *C*. *elegans* fed *E. coli* HB101 were starved for 12 h from Day 1 and refed with *E. coli* HB101 until quiescence behavior appeared, which was considered the satiated state (Fig. S2d).

### Oil Red O staining assays

Worms under normal conditions and fasting conditions (12 h) were washed with PBS and treated with fixation solution (2% paraformaldehyde) at -80 °C for 10 min. The worms were washed twice with PBS, resuspended in 60% isopropanol for 20 min and stained with 60% Oil Red O for 3 h (Soukas et al. 2009). Worms were washed with M9 buffer and decolorized with PBS solution containing 0.01% Tween. Then, worms were collected and imaged by microscopy.

### Lifespan

Lifespan was detected as described previously (Wang et al. 2022). Sixty nematodes at the L1 stage were cultured in NGM plates with OP50. The nematodes were transferred to new NGM plates when they grew to adulthood every day until all nematodes died. The number of dead nematodes was calculated.

## Supplementary Information

**Additional file 1: Fig. S1. **Confirmation of the fasting and satiation models. (a) Representative images of Oil Red O staining levels in *C. elegans* under normal conditions and fasting for 12 h. (b) Survival curves of *C. elegans* under normal conditions and fasting for 12 h. Data are shown as the mean ± SEM (data statistics from 105-115 nematodes), *n* = 3, ***p < 0.001 by the log-rank test. (c) Images of *C. elegans* in the satiated state at 0, 1, 10, and 20 s. **Fig. S2.** Research schematic of the precision redox map of* C. elegans *during fasting, refeeding and satiation. (a) Hyperion and Grx1-roGFP2 probes were stably overexpressed in two tissues (body muscle and neurons) of three organelles (cytoplasm, mitochondria and ER) in *C. elegans*. (b) Transgenic *C. elegans* with redox fluorescent probes. (c). The fasting and refeeding stress models. *C. elegans fed E. coli* OP50 on Day 1 were *starved* for 12 h (fasting model), and then the *starved C. elegans were refed with E. coli* OP50 for 1 h (refeeding model). (d) Satiation stress model. *C. elegans fed E. coli* HB101 on Day 1 were starved for 12 h, and then the *starved C. elegans were refed with E. coli* HB101 until the *C. elegans* remained stationary for 10 s and their pharynx stopped pumping.

## Data Availability

All data and materials are available in the paper and online supplemental files.
