# Supplementary material for: Exploring the precision redox map during fasting-refeeding and satiation in C. elegans
Source: Stress Biol. 2023 Jun 12;3(1):17. doi: 10.1007/s44154-023-00096-z (PMC10442001; doi:10.1007/s44154-023-00096-z)
Supplement: Supplementary file 1 — Additional file 1: Fig. S1. Confirmation of the fasting and satiation models. (a) Representative images of Oil Red O staining levels in C. elegans under normal conditions and fasting for 12 h. (b) Survival curves of C. elegans under normal conditions and fasting for 12 h. Data are shown as the mean ± SEM (data statistics from 105-115 nematodes), n = 3, ***p < 0.001 by the log-rank test. (c) Images of C. elegans in the satiated state at 0, 1, 10, and 20 s. Fig. S2. Research schematic of the precision redox map of C. elegans during fasting, refeeding and satiation. (a) Hyperion and Grx1-roGFP2 probes were stably overexpressed in two tissues (body muscle and neurons) of three organelles (cytoplasm, mitochondria and ER) in C. elegans. (b) Transgenic C. elegans with redox fluorescent probes. (c). The fasting and refeeding stress models. C. elegans fed E. coli OP50 on Day 1 were starved for 12 h (fasting model), and then the starved C. elegans were refed with E. coli OP50 for 1 h (refeeding model). (d) Satiation stress model. C. elegans fed E. coli HB101 on Day 1 were starved for 12 h, and then the starved C. elegans were refed with E. coli HB101 until the C. elegans remained stationary for 10 s and their pharynx stopped pumping. [file 44154_2023_96_MOESM1_ESM.docx]

**Exploring the Precision Redox Map during Fasting-Refeeding and Satiation in *C. elegans***

Xinhua Qiao^1,†^, Lu Kang^2†^, Chang Shi^1,3†^, Aojun Ye^1,3†^, Dongli Wu^2^, Yuyunfei Huang^1,3^, Minghao Deng^1,3^, Jiarui Wang^1,3^, Yuzheng Zhao^4,*^, Chang Chen^1,2,3,*^

^1^National Laboratory of Biomacromolecules, CAS Center for Excellence in Biomacromolecules, Institute of Biophysics, Chinese Academy of Sciences, Beijing 100101, China

^2^School of Basic Medical Sciences of Southwest Medical University, Luzhou, 646000, China

^3^University of Chinese Academy of Sciences, Beijing 100049, China

^4^School of Pharmacy, East China University of Science and Technology, Shanghai 200237, China

^#^Equal contribution.

^*^Correspondence authors: [changchen@ibp.ac.cn](mailto:changchen@ibp.ac.cn), yuzhengzhao@ecust.edu.cn


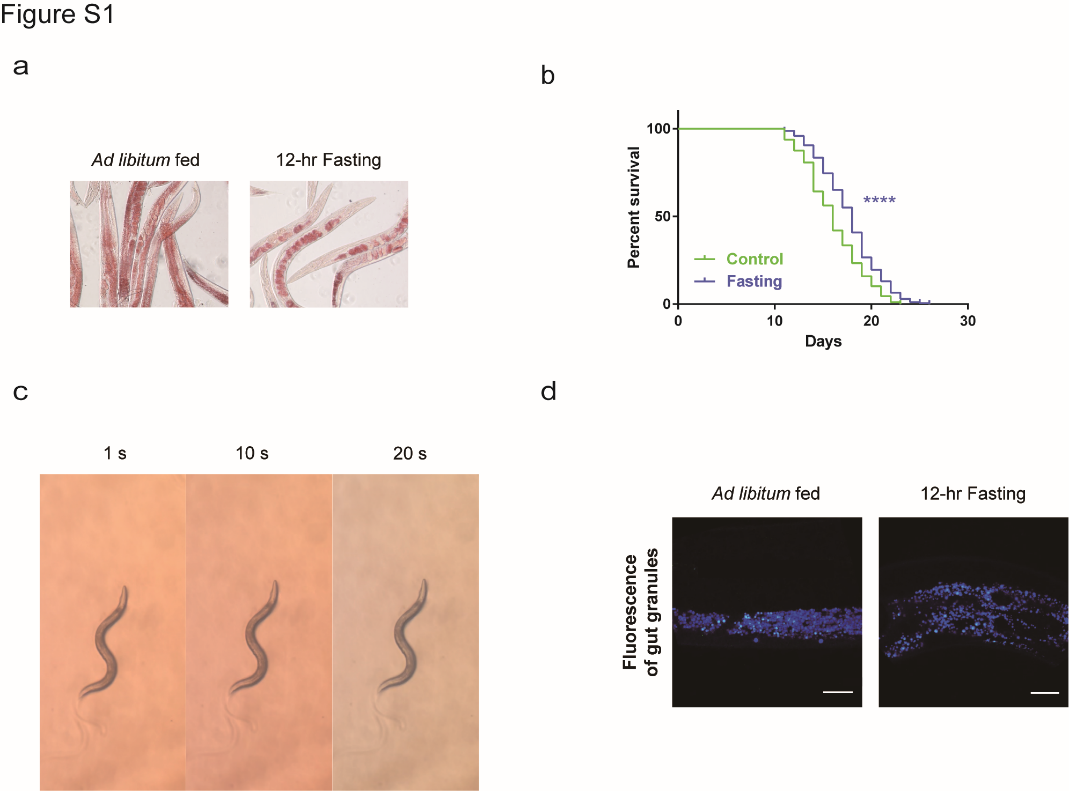


**Fig. S1. Confirmation of the fasting and satiation models.**

1. Representative images of Oil Red O staining levels in *C. elegans* under normal conditions and fasting for 12 hours. (b) Survival curves of *C. elegans* under normal conditions and fasting for 12 hours*.* Data are shown as the mean ± SEM (data statistics from 105-115 nematodes), n = 3, ****p* < 0.001 by the log-rank test. (c) Images of *C. elegans* in the satiated state at 0, 1, 10, and 20 seconds.


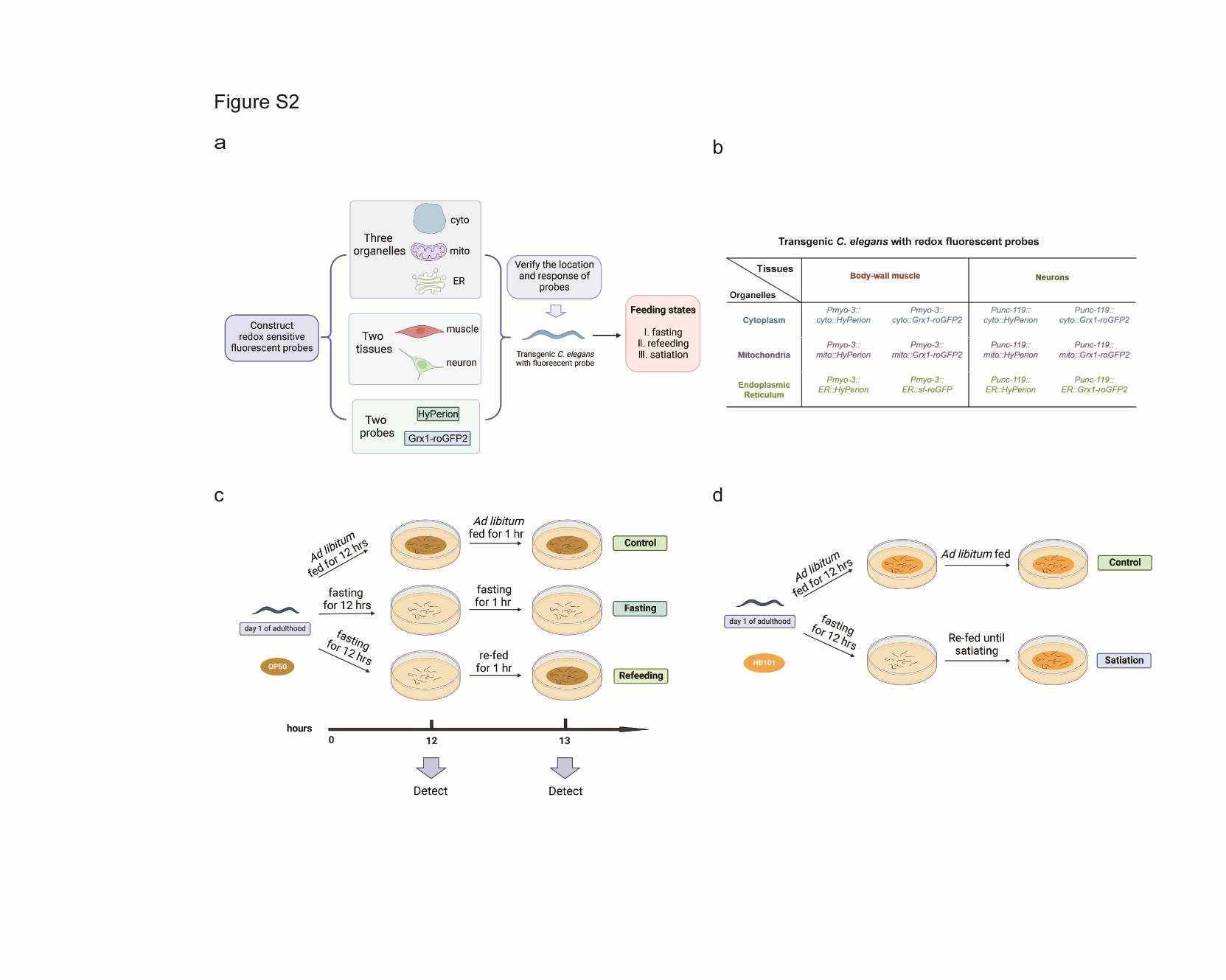


**Fig. S2. Research schematic of the precision redox map of *C. elegans* during fasting, refeeding and satiation.** (a) Hyperion and Grx1-roGFP2 probes were stably overexpressed in two tissues (body muscle and neurons) of three organelles (cytoplasm, mitochondria and ER) in *C. elegans*. (b) Transgenic *C. elegans* with redox fluorescent probes. (c). The fasting and refeeding stress models. *C. elegans* fed *E. coli* OP50 on Day 1 were starved for 12 hours (fasting model), and then the starved C. elegans were refed with *E. coli* OP50 for 1 hour (refeeding model). (d) Satiation stress model. *C. elegans* fed *E. coli* HB101 on Day 1 were starved for 12 hours, and then the starved C. elegans were refed with *E. coli* HB101 until the C. elegans remained stationary for 10 seconds and their pharynx stopped pumping.
